# Profiling the Proteome of Cyst Nematode-Induced Syncytia on Tomato Roots

**DOI:** 10.3390/ijms222212147

**Published:** 2021-11-10

**Authors:** Marcin Filipecki, Marek Żurczak, Mateusz Matuszkiewicz, Magdalena Święcicka, Wojciech Kurek, Jarosław Olszewski, Marek Daniel Koter, Douglas Lamont, Mirosław Sobczak

**Affiliations:** 1 Department of Plant Genetics, Breeding and Biotechnology, Institute of Biology, Faculty of Biology and Biotechnology, Warsaw University of Life Sciences, Nowoursynowska 159, 02-776 Warsaw, Poland; marek.zurczak@gmail.com (M.Ż.); Mateusz_Matuszkiewicz@sggw.edu.pl (M.M.); marek_koter@sggw.edu.pl (M.D.K.); 2 Department of Botany, Institute of Biology, Faculty of Biology and Biotechnology, Warsaw University of Life Sciences, Nowoursynowska 159, 02-776 Warsaw, Poland; magdalena_swiecicka@sggw.edu.pl (M.Ś.); wojciech_kurek@sggw.edu.pl (W.K.); miroslaw_sobczak@sggw.edu.pl (M.S.); 3 Veterinary Research Centre, Centre for Biomedicine Research, Centre for Regenerative Medicine, Department of Large Animal Diseases and Clinic, Institute for Veterinary Medicine, Warsaw University of Life Sciences, Nowoursynowska 100, 02-797 Warsaw, Poland; jaroslaw_olszewski@sggw.edu.pl; 4 ‘FingerPrints’ Proteomics Facility, College of Life Sciences, University of Dundee, Dow Street, Dundee DD1 5EH, UK; d.j.lamont@dundee.ac.uk

**Keywords:** *Globodera rostochiensis*, *Solanum lycopersicum*, syncytium, proteome, laser capture microdissection, mass spectrometry

## Abstract

Cyst nematodes are important herbivorous pests in agriculture that obtain nutrients through specialized root structures termed syncytia. Syncytium initiation, development, and functioning are a research focus because syncytia are the primary interface for molecular interactions between the host plant and parasite. The small size and complex development (over approximately two weeks) of syncytia hinder precise analyses, therefore most studies have analyzed the transcriptome of infested whole-root systems or syncytia-containing root segments. Here, we describe an effective procedure to microdissect syncytia induced by *Globodera rostochiensis* from tomato roots and to analyze the syncytial proteome using mass spectrometry. As little as 15 mm^2^ of 10-µm-thick sections dissected from 30 syncytia enabled the identification of 100–200 proteins in each sample, indicating that mass-spectrometric methods currently in use achieved acceptable sensitivity for proteome profiling of microscopic samples of plant tissues (approximately 100 µg). Among the identified proteins, 48 were specifically detected in syncytia and 7 in uninfected roots. The occurrence of approximately 50% of these proteins in syncytia was not correlated with transcript abundance estimated by quantitative reverse-transcription PCR analysis. The functional categories of these proteins confirmed that protein turnover, stress responses, and intracellular trafficking are important components of the proteome dynamics of developing syncytia.

## 1. Introduction

Sedentary plant-parasitic nematodes (PPNs) are important soilborne pests of most crops worldwide. After root invasion, PPNs induce root cells to develop into specialized feeding structures termed syncytia or giant cells, which are characteristic for cyst-forming nematodes (genera *Heterodera* and *Globodera*) and root-knot nematodes (genus *Meloidogyne*), respectively. These specific structures become the sole food source for developing juveniles and adult females. The syncytia are established by stimulation of the partial dissolution of the cell walls between a group of hypertrophied parenchymatous vascular cylinder cells by the infective juveniles [1]. Thus, syncytium formation is accompanied by substantial developmental and metabolic localized reprogramming of root cells [2,3,4,5], as well as by systemic changes in the entire plant body [6]. The observed localized and systemic plant reactions to nematode parasitism comprise a sequence of overlapping, specific responses to root invasion by juveniles, their migration through plant tissues, then modification of the selected initial syncytial cell, syncytium expansion, and syncytial functioning, all triggered by mechanical, biochemical, and genetic stimuli. 

Recently, considerable research attention has focused on the parasite-derived effectors secreted by the nematode cuticle and esophageal glands, which facilitate host recognition, root invasion, and further syncytium development in parallel with suppression of plant defense responses [7,8,9,10,11,12,13]. Although proteins are crucial constituents of nematode secretions and markers for syncytium development, current knowledge is derived mostly from transcriptomic analyses, which have detected hundreds of putative nematode effectors and thousands of deregulated plant genes [3,4,10,12,14]. These transcriptomic analyses have revealed that specific functional gene categories, such as “protein biosynthesis” (e.g., elongation factors and chaperonins), “protein modification” (e.g., kinases and phosphatases), and “protein degradation” (e.g., metacaspases and ubiquitin ligases), are important components of the gene-expression dynamics [15,16,17]. Moreover, microtranscriptomic data indicate that miRNA-mediated negative regulation at the translational level complicate the simplified transcriptomic view of plant responses to nematode infections [18]. Thus, the study of plant–nematode interactions at the protein level is attractive but is faced with a number of technical obstacles, which may be generally defined as low throughput.

The first paper to describe plant–nematode interaction at the protein level was published by Hammond-Kosack and co-workers in 1990 [19]. These authors studied proteins originating from in vitro translation of mRNA isolated from *G. rostochiensis*-infected potato plants carrying the *H1* resistance gene. Callahan et al. used two-dimensional polyacrylamide gel electrophoresis (2D-PAGE) to study proteins isolated directly from infected and uninfected roots of susceptible and resistant cultivars of cotton [20]. The authors identified a novel 14 kDa polypeptide for which expression was induced by the root-knot nematode, *M. incognita*. More recently, Hütten et al. performed activity-based protein profiling to reveal intriguing variation in protein activity in *Arabidopsis thaliana* roots infected with *H. schachtii* [21]. The results revealed, for example, differential activity of S-formyl-glutathione hydrolase, methylesterase, and vacuolar processing enzymes. 

In contrast to transcriptomic analyses, precise and comprehensive analyses of changes in the syncytial proteome remain rare. This situation may change soon as, given the improving resolution of mass spectrometry (MS), high-throughput protein identification will become a realistic tool to supplement existing data and provide novel information. Such proteomic studies have been conducted on plant–microbe interactions; for a review see [22]. For example, changes in the proteome of vesicular-arbuscular mycorrhizae were analyzed using two-dimensional liquid chromatography and tandem mass spectrometry (2D-LC-MS/MS). Eighty-seven proteins were identified, of which two were differentially expressed [23]. Application of sensitive MS methods to study plant responses to infection by pathogenic fungi enables precise characterization of many proteins for which traditional molecular methods are insufficiently sensitive. For example, a novel member of the pathogenesis-related group 5 (PR5) protein family was identified in tomato xylem sap after infection with the vascular wilt fungus *Fusarium oxysporum* [24]. Wheat infected with *F. graminearum* has been comprehensively investigated using proteomic methods; 30 [25] and 41 [26] differentially regulated proteins were identified, including pathogenesis-related (PR) proteins, as well as proteins involved in the antioxidant and jasmonic acid signaling pathways. 

An additional advantage of using MS in analyses of plant–pathogen interactions is its ability to detect post-translational protein modifications (PTMs). Together with the control of enzyme activity, transcriptional events, cell division, and subcellular protein localization, PTM by phosphorylation/dephosphorylation is a general mechanism in the perception and transduction of pathogen-derived signals, as well as a crucial regulatory process in effector-stimulated plant defense responses [27]. A comprehensive phosphoproteome map has been determined for *Arabidopsis* cell cultures in response to bacterial and fungal elicitors [28]. Protein phosphorylation patterns are specific to elicitors; for example, AtPhos43 is phosphorylated when exposed to bacterial flagellin, but not when exposed to fungal chitin [28]. 

Although the effect of cyst nematode parasitism is the formation of syncytia in infected roots, comparison of independent molecular analyses is often ambiguous owing to disparate tissue sampling methods and experimental conditions. Nucleic acids, proteins, or metabolites are usually isolated from entire root systems containing several syncytia or from root segments composed of syncytia and surrounding non-modified cells. Thus, the isolated samples are enriched in differentially and specifically produced molecules and represent a mixture of systemic and local reactions. Such analyses are prone to errors associated with the relative proportions of healthy and infected tissues, total number of simultaneous infections per root system, and presence or absence of root apical meristems, for example. To improve the uniformity and quality of analyzed samples, enrichment in purified material originating from the syncytial protoplasts is desirable. Such localized specificity of the sample can be achieved by microaspiration of the syncytial cytoplasm [5] or laser-capture microdissection (LCM) of syncytia from infected root sections [29,30,31,32]. In particular, given its relative ease and reliability, an increasing number of successful biological applications of LCM has allowed isolation of RNA, DNA, proteins, and metabolites from heterogeneous populations of cells [33,34]. The technical principle of LCM is based on laser-cutting isolation of the microscopically identified cells from microtome-sectioned samples and scratching them from a support. When a plastic membrane is used as a support, it is cut together with the selected tissue fragment. Advantages of LCM are the excellent morphological and biochemical preservation and recognition of tissues to ensure the high specificity of isolated samples. Nevertheless, LCM of plant tissues is especially challenging owing to the rigid cell wall and highly vacuolated protoplast.

In plant biological research, LCM is used mostly for transcript profiling by microarrays or RNA-sequencing and for more precisely targeted expression analysis by quantitative reverse-transcription PCR (RT-qPCR). Given the strong potential and versatility of LCM, the procedure has been applied previously in plant–nematode interaction research to study plant responses to infection by cyst nematodes [29,30,31,32,35] and root-knot nematodes [36,37,38]. 

To the best of our knowledge, no protocol incorporating MS analysis of proteins isolated from nematode-induced plant syncytia has been published to date. In this paper, we describe an optimized procedure for LCM of syncytial cells from tomato roots and subsequent protein analysis using MS. Proteome profiling is an effective strategy for gene discovery in studies of plant–nematode interactions.

## 2. Results

### 2.1. Optimization of the Fixation Method, Tissue Embedding, and Sectioning Procedure

To isolate syncytial proteins from *G. rostochiensis*-induced syncytia, tomato seedlings were cultured on Petri dishes containing KNOP medium for two weeks and infected with J2s of the nematode. Root segments containing syncytia were dissected at 7 dpi. In any analytical procedure involving microscopy, maintenance of cytological and morphological fidelity is extremely critical. Samples for immunohistological analyses are usually fixed in a formaldehyde-containing fixative, whereas ethanol–acetic acid solutions are preferred for fixation in experiments involving RNA extraction [29,39]. With subsequent proteomic analyses in mind, we compared the method without fixation or with relatively mild fixation (4% paraformaldehyde). After fixation, tissues are routinely dehydrated and embedded either in paraffin for cutting at room temperature or in tissue fixation medium (TFM), or another cryoprotection medium, for cryosectioning. Irrespective of which embedding and sectioning method was used, in the case of nematode-induced root syncytia, histological and anatomical interpretation was hindered by the irregular shape of the feeding structure and root curvature. To overcome these obstacles and to collect reasonable amounts of syncytial material, the use of syncytia at 7 dpi was optimal. For preservation of protein integrity, cryosectioning was optimal because of reduction in the number of processing steps. Additionally, the embedding in paraffin must be avoided as it might interfere with subsequent LCM using recommended PET slides for sections mounting (deparaffinization in xylene dissolves polyethylene).

**Figure 1:**
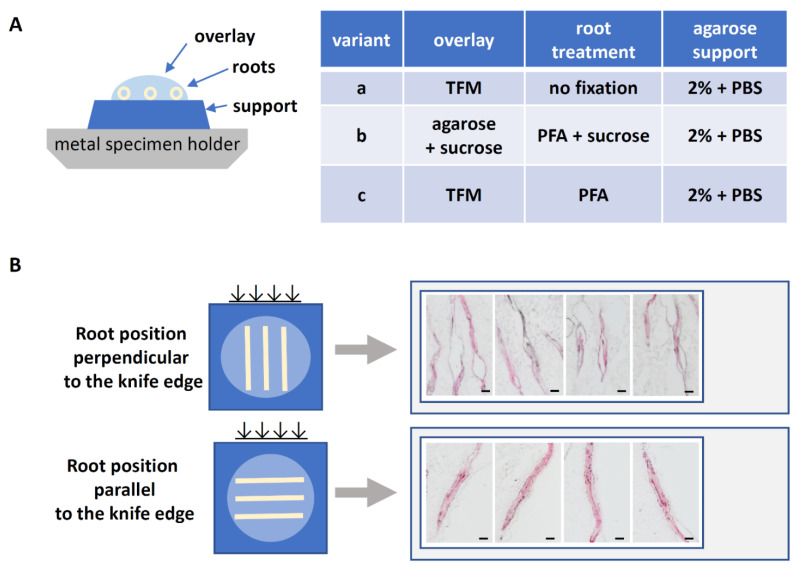

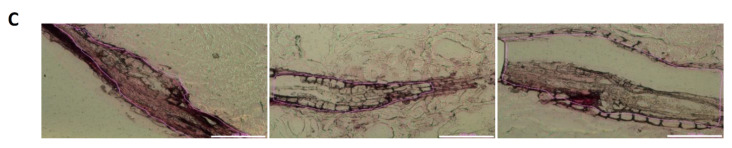
Optimization steps for cryosectioning of tomato syncytia before microdissection. (**A**) Organization of root segments on the specimen holder and the three variant procedures for fixation and mounting of the samples. (**B**) Effect of root segment orientation in relation to the knife blade on the section quality. (**C**) Morphology of sections generated with fixation variant b and longitudinally sectioned (roots oriented parallel to the knife edge). Violet lines outline the dissected regions. Abbreviations: TFM, tissue freezing medium; PBS, phosphate buffered saline; PFA, paraformaldehyde fixative. Scale bars in (**B**,**C**): 200 µm.

After selection of syncytia at 7 dpi, which provided the longest and largest continuous sections for subsequent procedures, different fixation and cryoprotection methods were evaluated to obtain the best-quality samples with regard to sample anatomy and ultrastructure. Three divergent sample preparation procedures were compared (Figure 1). We prepared frozen specimens without fixation and cryoprotection (variant a, Figure 1A) as well as specimens fixed in PFA and cryoprotected in sucrose or TFM (variants b and c, Figure 1A). Treatment with 34% sucrose successfully substituted polyvinyl alcohol, polyethylene glycol, and resins present in the TFM without noticeable effects (the polymers present in the TFM medium may give nonspecific, repetitive background signals on mass spectra). We opted to use longitudinal sectioning and thus we combined five or six root segments (containing syncytia or uninfected controls) into a single specimen to be sectioned concurrently (Figure 1B). To mount root segments within a specimen, we used an overlay of either TFM medium (variant c, Figure 1A) or 1.5% (*w*/*v*) agarose gel in 34% (*w*/*v*) sucrose solution (variant b, Figure 1A). The sections were cut with a CM1860 Cryostat. Several thicknesses of sections were tested. Only 10-µm-thick sections provided an acceptable quality and structure. The optimization of fixation, cryoprotection, and sectioning yielded a satisfactory tissue quality that allowed discrimination of the root mesophyll and syncytia. The only drawback was dissociation of tissue layers visible in the longitudinal sections, which was minimized when the cryostat knife edge was oriented parallel to the root (Figure 1B,C).

### 2.2. Tissue Analysis and LCM

For LCM, a Leica LMD7 laser microdissection microscope was used. Optimization of LCM was focused on adjustment of the laser light intensity and pulse frequency. Increasing the pulse frequency from 120 to 500 and decreasing the offset from 110 to 50 resulted in rapid and effective collection of samples for protein isolation and MS analysis. 

### 2.3. Protein Isolation

When collecting dried samples microdissected from sections mounted on PET film slides, it was critical to transfer them immediately to the protein solubilization buffer containing 8 M urea and protease inhibitors cocktail (Table 1). The collection step should not be longer than 30 min owing to water evaporation from the collection tubes and crystallization of urea. The homogenization step also greatly improved the protein recovery rate, with the best results obtained when an Eppendorf tube pestle was used compared with glass beads in a homogenizer. After centrifugation, proteins in the residue were precipitated with methanol–chloroform or proteins were directly separated by PAGE. The precipitated protein samples yielded a higher amount of peptides detected by MS. After trypsin digestion, the peptide samples were analyzed by liquid chromatography to examine unidentified regular repetitive spectral peaks possibly caused by the polymer residue background. Therefore, an additional protein purification step was applied.

### 2.4. Mass Spectrometry Results

**Figure 2:**
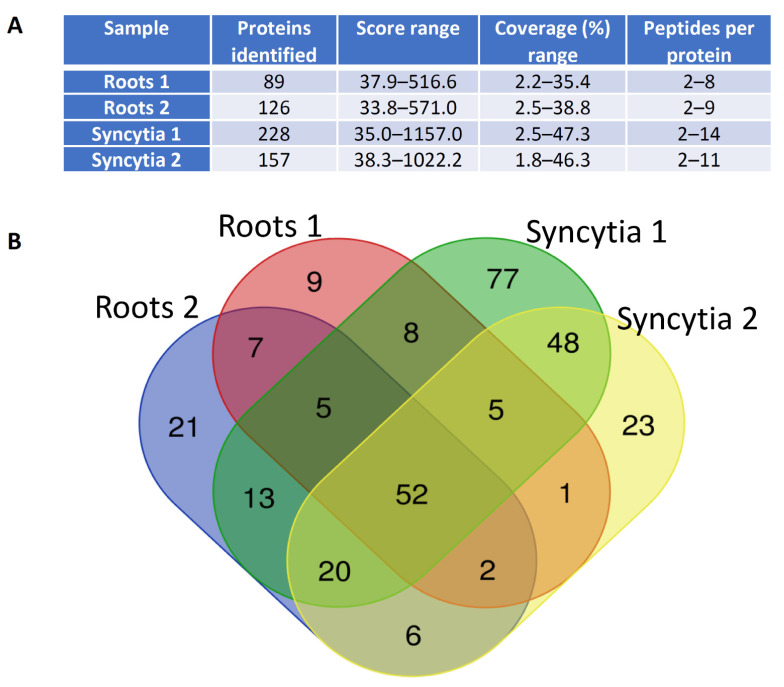
Mass spectrometric analysis of isolated proteins. (**A**) Sample data. (**B**) Venn diagram of proteins identified in all samples. Proteins detected repeatedly and specifically in both samples, seven root-specific proteins, and 48 syncytium-specific proteins are listed in Table S1. We also identified seven proteins absent or downregulated in samples of syncytia, including plasma membrane intrinsic protein 2.6, vacuolar α-mannosidase, and xyloglucan endoglucanase inhibitor (Table S1). The first two proteins are possibly involved in regulation of turgor pressure and the third protein participates in cell wall remodeling.

Using samples containing approximately 5 µg total protein, we were able to detect 100–200 proteins per sample (Figure 2A). Ultimately, we identified 48 proteins detected solely in samples of syncytia, and seven proteins specific to uninfected root cells (Figure 2B, Table S1).

**Figure 3:**
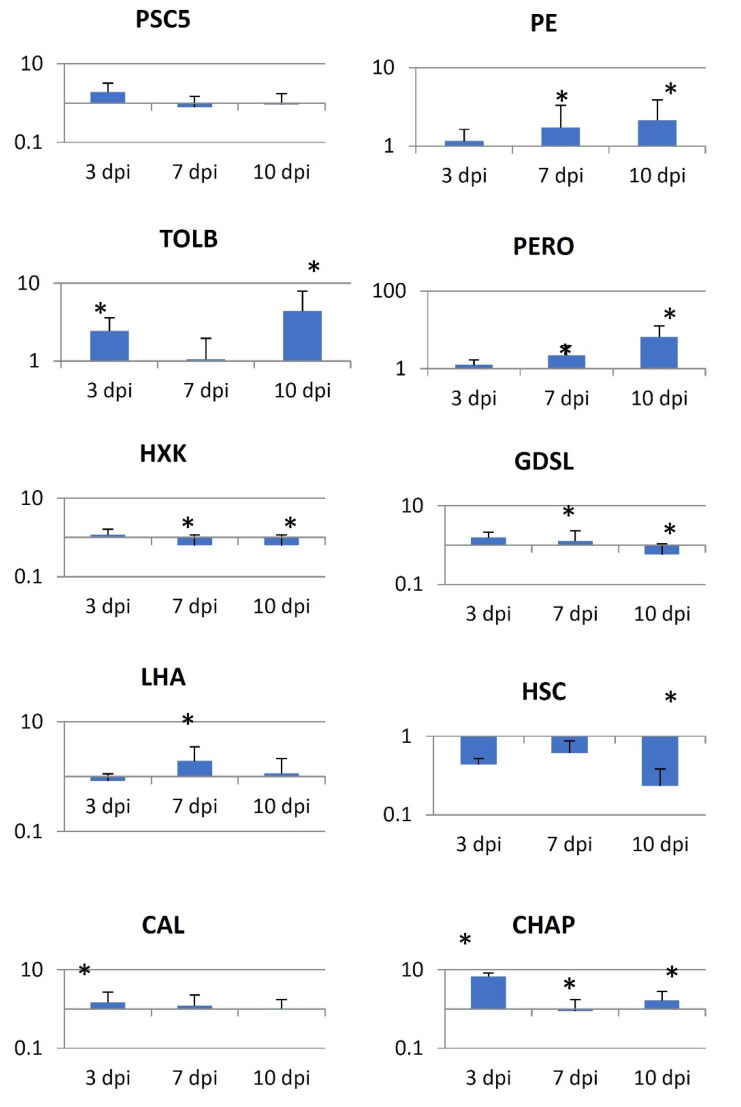
Results of RT-qPCR analysis of selected tomato transcripts at 3, 7, and 10 days post-inoculation (dpi) in syncytia to verify proteomic variation. PSC5—PAD1 (Solyc01g111450) proteasome, alpha subunit; PE—ASCO (Solyc04g082140) L-ascorbate oxidase homolog, pectinesterase; TOLB (Solyc06g008620) tolB protein-like protein; PERO (Solyc05g046010) peroxidase; HXK—HXK1 (Solyc03g121070) hexokinase; GDSL (Solyc12g017460) GDSL esterase/lipase; LHA—LHA2 (Solyc06g071100) plasma membrane ATPase; HSC—HSC2L (Solyc09g010630) heat shock cognate 70 kDa protein 2; CAL—CAM1 (Solyc01g008950) calmodulin 1; CHAP—CPN60B2 (Solyc01g028810) chaperonin-60 beta subunit. * *p* ≤ 0.05.

The large proportion of syncytia-specific proteins (11, Table S1) was associated with protein biosynthesis and degradation, which was consistent with substantial developmental and metabolic reprogramming of syncytia. This group of proteins included two **proteasome α-subunit components** (expressed proteins highlighted in bold were also verified at the transcript levels using RT-qPCR; Figure 3), proteasome regulatory protein, three ribosomal proteins, two elongation factors, and protein phosphatase. Seven proteins specific to syncytia were associated with stress responses: **HSC2-like protein**, which is a HSP70 homolog, **chaperonin-60 β-subunit**, calnexin (a calcium-binding protein), **calmodulin 1**, **GDSL esterase/lipase**, and two PR proteins. Five syncytia-specific proteins were associated with redox reactions and may be alternatively classified as stress responsive or involved in general metabolism. This group included three **peroxidases**, catalase, and an **L-ascorbate oxidase** homolog. An additional intriguing group contained three intracellular trafficking proteins: clathrin heavy chain, Ran binding protein-1, and **tolB protein-like**. **Hexokinase**, osmotin, and dehydrin may contribute to the increase in turgor pressure observed in syncytia [40]. A group of proteins involved in numerous general cellular and physiological processes were detected, but their classification may be somewhat confusing, especially in relation to syncytium development and functioning (e.g., **plasma membrane ATPase**).

An important question was how the abundance of these proteins correlated with the corresponding transcript levels in syncytia. We selected 10 syncytium-specific proteins detected by MS and estimated their transcript levels in root segments containing syncytia at 3, 7, and 10 dpi. Interestingly, only five genes showed transcriptional upregulation, whereas two genes were clearly downregulated and three showed no change or inconclusive changes in either direction (Figure 3).

To further explore the transcriptional/translational discrepancies, we evaluated the correspondence between the tomato proteomic data and the transcriptome of syncytia induced in *Arabidopsis* roots by *H. schachtii*, a model system for plant–cyst nematode interaction research. We selected a group of putative *Arabidopsis* orthologs of identified tomato proteins (Table S2) and examined their transcriptional profile in the Nematic database [41]. The Nematic database contains data generated by several plant–nematode interaction transcriptomic studies, including a dataset based on transcriptome profiles obtained from microaspirated syncytial protoplasts at 5 and 15 dpi [5]. Of the selected putative orthologs, 62% showed no change in transcript level, 32% were upregulated, and 6% were downregulated. 

## 3. Discussion

Previous reports on the application of LCM for plant molecular study involved mainly transcriptomic analyses; for example [42,43]. These studies include research on plant–nematode interactions in soybean [29,30], rice [38], tomato [36,44], and *Arabidopsis* [32,37]. However, no protocol for analysis of proteins isolated from syncytial cells has been published previously. We present here a procedure for optimization of the collection of plant tissue samples for proteomic analysis using LCM technology and demonstrate its application for comparative proteome analyses during the development of cyst nematode-induced syncytia in tomato roots. This procedure may provide answers to two basic research questions: first, whether even small amounts of syncytial tissue samples are sufficient for proteomic analyses, and second, to what extent the proteomic changes overlap with transcriptomic changes on which most analyses of plant–nematode interactions are based. The present study provides an affirmative answer to the first question and presents data to elucidate the second question.

The ability to collect an adequate amount of microdissected tissue represents a bottleneck for the proposed procedure. Assuming that the area of an average tomato syncytium at 7 dpi is 0.1 mm^2^, each sample requires 150 good-quality root sections (generated by serial sectioning of 30 syncytia) to attain a total syncytial area of 15 mm^2^, which will yield 5 µg protein. A realistic plan to monitor more than 1000 proteins would require considerably more than 10-fold of the protein sample used in the present study, which will make the procedure highly laborious and expensive. Thus, further technological advances are required to achieve this scale of monitoring. Nevertheless, even the fragmentary results presented here are extremely valuable given the present scarcity of such data and the results revealed the translational effects of cellular reprogramming in syncytia. We observed a distinct discrepancy between transcript and protein changes in syncytia for as much as 50% of the examined genes. It is not a surprise, but it implies that extreme caution is required when drawing conclusions from transcriptome-based results. Caution is even more justified when subsequent regulatory levels are taken into account, e.g., post-translational modifications, subcellular localization, or substrate availability. In addition, the substantial number of detected proteome components involved in protein degradation, biosynthesis, and cellular trafficking processes supports not only the known enhancement of metabolic activity of syncytia, but also more recent findings on the role of autophagy and organellar de novo biogenesis during plant–nematode interactions [15,45].

## 4. Materials and Methods

### 4.1. Plant Material and Specimen Preparation

Seedlings of the *Globodera rostochiensis*-susceptible tomato (*Solanum lycopersicum* L.) ‘MoneyMaker’ were aseptically grown on Petri dishes as described previously [46]. The seedlings were inoculated with infective second-stage juveniles (J2s) of *G. rostochiensis*. Samples comprising a segment of root containing a nematode-induced syncytium and attached juveniles were collected at 7 and 10 days post-inoculation (dpi). To optimize the cryosectioning procedure, three sample preparation procedures were tested (Figure 1A). In the first procedure, samples were directly frozen in liquid nitrogen without prior fixation (variant a, Figure 1A). In the other two procedures (variants b and c, Figure 1A), samples were fixed in 4% (*w*/*v*) paraformaldehyde dissolved in 0.1 M PBS (PFA) at room temperature for at least 2 h, including 30 min infiltration under 0.4 MPa vacuum. After washing in PBS at room temperature for 5 min, the fixed samples were stained with 1% (*w*/*v*) safranin-O in PBS for 10 min and then washed three times in PBS for 5 min. The samples then were divided into two batches: one was gradually cryoprotected with 15% (*w*/*v*) sucrose in PBS at 4 °C for 4 h and then in 34% (*w*/*v*) sucrose in PBS at 4 °C overnight (variant b, Figure 1A), whereas in variant c the cryoprotection step was omitted. For sectioning, a group of five or six root fragments were placed on a metal holder coated with a 5-mm-thick layer of solidified 2% (*w*/*v*) agarose and embedded in an overlay of tissue freezing medium (TFM; Leica, Wetzlar, Germany) or in 1.5% (*w*/*v*) agarose in 34% (*w*/*v*) sucrose dissolved in PBS (Figure 1A). The mounted samples were frozen in liquid nitrogen and stored at −80 °C. Longitudinal sections of thickness ranging between 2 and 10 µm were cut using a CM1860 Cryostat (Leica, Nussloch, Germany) operating at −30 °C parallel or perpendicular to the knife edge (Figure 1B). The sections were mounted on Leica PET Frame Slides (Leica, Wetzlar, Germany) dedicated for proteomic analyses. 

### 4.2. Laser-Capture Microdissection and Protein Sample Preparation

To dissect syncytial tissue, a Leica LMD7 laser microdissection microscope (Leica, Wetzlar, Germany) was used with the pulse frequency set at 500 and offset at 50. The precise dissection was done at 400× magnification. The dissected tissue fragments together with the underlying PET film were collected directly in tubes containing extraction buffer (100 mM Tris [pH 8.5], 150 mM NaCl, 5 mM EDTA, 10 mM DTT, 0.5% Triton X-100, protease inhibitors cocktail, PhosSTOP phosphatase inhibitors, and 8 M urea). After grinding with a tube-fitted pestle and centrifugation of the solid residues, the proteins were methanol–chloroform precipitated and submitted to the proteome analysis.

Samples were resuspended in 100 µL of 100 mM ammonium bicarbonate, vortexed prior to addition of 1ul of 1M DTT and incubated for 30 minutes at 50 °C. Then the samples were cooled prior to the addition of 2 µL of 1M iodoacetamide and afterwards incubated in the dark at room temperature for 30 minutes. Quenching of the reaction was done by addition of 1 µL of 1M DTT. Subsequently, the samples were digested with 0.5 µL of trypsin (1 µg/µL) for 5 h at 37 °C, and then 0.5 µL (1 µg/µL) aliquot of trypsin added for overnight digest. After, digestion samples were resuspended in 10 µL of 5% formic acid and diluted to 50 µL using milliQ water. To remove polymer contamination, Hippr columns (Thermo Fisher Scientific, Waltham, MA, USA) were used.

### 4.3. Mass Spectrometry

Analysis was performed by FingerPrints Proteomics Facilities at the University of Dundee. Peptide mixtures were analyzed by liquid chromatography coupled to tandem mass spectrometry (LC-MS/MS) using an Ultimate 3000 RSLCnano system (Thermo Fisher Scientific, Waltham, MA, USA), online connected to a LTQ Orbitrap Velos Pro (Thermo Fisher Scientific, Waltham, MA, USA). LC buffers were made up to the following: Buffer A (0.1% formic acid in Milli-Q water (*v*/*v*)) and Buffer B (80% acetonitrile and 0.08% formic acid in Milli-Q water (*v*/*v*)). The peptides were initially trapped on an Acclaim Pepmap100 analytical column (C18, 100 µM × 2 cm) and separated on an Easy-Spray PepMap RSLC C18 column (75 µM x 50 cm; Thermo Electron Corp., San Jose, CA, USA). A program for gradient separation was used (mobile phase A: 0.1% FA in Milli-Q water; mobile phase B: 0.08% FA in 80% acetonitrile, flow rate 0.3 µL/min). The gradient elution started at 2% (0–5 min) of mobile phase B, increased from 5 to 35% (6–130 min), then increased to 98% (130–152 min) of mobile phase B, then returned to 2% (153 min) and remained at this state for the next 20 min.

Samples were transferred to a mass spectrometer via an Easy-Spray source with the temperature set at 50 °C and a source voltage of 1.9 kV. MS data were acquired in a data-dependent strategy selecting up to the top 20 precursors based on precursor abundance in the survey scan (*m*/*z* 335–1800).

The acquired raw data were processed using Mascot Search Engine 2.4.1 (Matrix Science, London, UK) and then searched against the ITAG3.0 (International Tomato Genome Sequencing Project) tomato genomic database using the Mascot search engine. The search parameters were as follows: precursor mass tolerance, 10 ppm; fragment mass tolerance, 0.6 Da; enzyme specificity, trypsin/P; maximum missed cleavage sites allowed, 2; static modification, carbamidomethyl (C); dynamic modification: oxidation (M), dioxidation (M), acetyl (N-term), Gln- > pyro-Glu (N-term Q), phospho (ST; Y).

Peptides with a Mascot score exceeding the threshold value corresponding to Target FDR (Strict): 0.01 and Target FDR (Relaxed): 0.05 were considered to be positively identified.

### 4.4. RNA Isolation, cDNA Synthesis, and qPCR Reaction

The GeneMATRIX Universal RNA Purification Kit (version 1.2) (Eurx, Gdańsk, Poland) was used to extract total RNA from nematode-infected and uninfected control tomato roots in accordance with the manufacturer’s protocol. The RNA integrity and concentration were measured with a NanoDrop 2000 spectrophotometer. A sample (1 µg) of total RNA was transcribed to cDNA using the QuantiTect Reverse Transcription Kit (Qiagen, Hilden, Germany) in accordance with the manufacturer’s protocol. 

Real-time PCR experiments were conducted in 96-well reaction plates using a Bio-Rad CFX96 Touch™ Real-Time PCR Detection System (Bio-Rad, Hercules, CA, USA). The reaction conditions were as follows: denaturation at 95 °C for 3 min, and 40 cycles of 95 °C for 10 s and 60 °C for 30 s. The reaction mixture (total volume 20 µL) comprised 8 μL cDNA (2.5 ng/μL), 1 μL each gene-specific primer (10 mM), and 10 μL 2× Ready Fast Green Mix reagent (Biochem Development, Gdańsk, Poland). Two tomato genes, SAND (SGN-U316474) and RPL8 (NM_001247186), were used as internal reference genes. The transcript level of the selected genes was normalized to that of *SAND* and *RPL8* using the 2^−ΔΔCt^ method [47]. Statistical analysis was performed using the REST tool [48]. The sequences of all primers used in the analysis are presented in Table S3.

## Figures and Tables

**Table 1:** Summary of protein purification steps tested for optimal recovery of proteins from laser-capture-microdissected samples. The optimal procedure is in the last row.

Collection Buffer	Tissue Homogenization	Protein Sample Preparation	Polymer Purification	Sample Size
8 M urea, 1× protease inhibitors cocktail, 1× phosphatase inhibitors cocktail	Homogenizer and glass beads	PAGE-separated gel sections	Not included	4 mm^2^
8M urea, 1× protease inhibitors cocktail, 1× phosphatase inhibitors cocktail, 100 mM Tris, 150 mM NaCl, 5 mM EDTA, 10 mM DTT, 0.5% (*v*/*v*) Triton X-100	Pestle and Eppendorf tube	Methanol–chloroform precipitate	Included	15 mm^2^

## Data Availability

Data is contained within the article or Supplementary Material.

## Supplementary Materials

The following are available online at https://www.mdpi.com/article/10.3390/ijms222212147/s1.
