# Supplementary material for: Profiling the Proteome of Cyst Nematode-Induced Syncytia on Tomato Roots
Source: Int J Mol Sci. 2021 Nov 10;22(22):12147. doi: 10.3390/ijms222212147 (PMC8625192; doi:10.3390/ijms222212147)
Supplement: Supplementary file 1 [file ijms-22-12147-s001.zip › Table S3 Primers used in the qRT-PCR reactions.pdf]

Table S3. Primers used in the qRT-PCR reactions.

Gene	Sequences (5' – 3')
Acid phosphatase	F: CGATGTCGCTTTTGGGTCGATA<br>R: TGGTGCTTTTCCTTCTCTCGT
lipid desaturase; cevi19	F: TCAAGGTTGTGTTTGCACCG<br>R: CCTGTGTTTGCATGGTGACG
trans-cinnamate 4-monooxygenase, cyp73A	F: GCTTAGACCATTCTTGAGAGGG<br>R: CCATTTTCAGCCATTAGTTTCCTTC
cytochrome P450	F: ACGATTGCAGGAAAAAGGTACT<br>R: CACCAACAGCTTCAACGCC
defensin protein, DEFL1	F: TGCTTGTTATGGCTACTGAAATGG<br>R: ACGATGGCTCAACGACTCAC
Deoxyhypusine synthase	F: GCCAAGACTGTGAAGGTGCATT<br>R: ATATGTGTGGTCGGAAGGACA
Glutathione transferase	F: ATGCAAGAAAGACATGGGCA<br>R: TCCATACACCTTTTCACCCATGA
Isocitrate lyase, LEU18678	F: CTGCTGCTATGGGAAAAGGTGT<br>R: TTTGTTGCCCCAGTCCTTGT
Major latex-like protein	F: TGGAGGTTAAGTGTGGAGGAC<br>R: TAACCTTAACTTTTCCATCATGGGT
organ-specific protein S2-like	F: TCCATGCACCAAGCAAATATCAA<br>R: TGCTGCTACCATACAAGGCAA
Stress responsive A/B barrel domain family protein	F: TTGTCAAGTCATTTGTTTGGGGAA<br>R: CAGTGGCAAAAGTGGCTGAA
SAND	F: TTGCTTGGAGGAACAGACG<br>R: GCAAACAGAACCCCTGAATC
RPL8	F: CCGAAGGAGCTGTTGTTTGTA<br>R: ACCTGACCAATCATAGCACGA
